# A Cross-Sectional Clinical Investigation of Organisms Causing Vaginal Discharge in Patients in Rural Tamil Nadu, India

**DOI:** 10.7759/cureus.33979

**Published:** 2023-01-19

**Authors:** Nithya Priyadharshini Shanmugam, Amutha Balasundharam, Irene N Thomas, Radhakrishnan A., Joseph Jenson James

**Affiliations:** 1 Dermatology, Shri Sathya Sai Medical College and Research Institute, Shri Balaji Vidyapeeth, Chennai, IND; 2 Dermatology, Government Mohan Kumaramangalam Medical College and Hospital, Salem, IND; 3 Community Medicine, Nagpattinam Government Medical College, Nagapattinam, IND

**Keywords:** abnormal vaginal discharge, co-morbidities, high risk sexual behavior, candida, trichomoniasis, bacterial vaginosis

## Abstract

Background and aims

Abnormal vaginal discharge is a prevailing gynecological problem among women in the reproductive age group. Vaginal discharges have multiple etiologies, and the present study was conducted with the objective of determining the prevalence of common organisms causing vaginal discharge and correlating with its various types of clinical presentations in those women attending a rural health centre of a medical college in Tamil Nadu, India.

Materials and methods

The study was a cross-sectional descriptive study, conducted in a rural health center of a teaching hospital in Tamil Nadu, India, from February 2022 to July 2022. All the patients clinically having the symptoms of vaginitis and with a discharge were included in this study, and postmenopausal women and pregnant women were excluded. Data was collected from a total of 175 patients.

Results

The mean (SD) age of the study population was 34.8 (6.9) years. Almost half, 91 (52%), of the study participants were in the age group of 31-40 years. Bacterial vaginosis was found in 74 (42.3%) and was the most common cause of abnormal vaginal discharge in our study participants, followed by vulvovaginal candidiasis, 34 (19.4%). There were significant associations between high-risk sexual behavior and the presence of co-morbidities with abnormal vaginal discharge.

Conclusion

The most common causes of abnormal vaginal discharge were found to be bacterial vaginosis followed by vulvovaginal candidiasis. The study results help to initiate early appropriate treatment for effective management of a community health problem.

## Introduction

Women in the reproductive age range frequently have abnormal vaginal discharge, which has a variety of etiologies. Abnormal vaginal discharge is the second most frequent issue, behind menstrual problems [1]. Over the course of a year, about 1 in 10 women will have vaginal discharge [2,3]. Each year, an estimated 10 million women visit primary care centres with complaints of excess or abnormal vaginal discharge. Many women who experience vaginal discharge mistreat their condition by taking over-the-counter medications [4]. If the right laboratory tests are not performed at the right time, medical professionals themselves are at risk of making an incorrect diagnosis

Physiological vaginal discharge alters with the menstrual cycle with the discharges being clearly pliable in consistency around ovulation and then becoming heavy and mild yellow during the luteal phase. Typical vaginal discharges are not associated with symptoms such as itching, redness and swelling, nor have an odour. An increased amount of discharge is seen during elevated oestrogen states such as ovulation, the luteal phase, puberty and pregnancy. Oestrogen-based therapies that combine hormonal contraception and hormone-replacement therapies also play a major role.

Depending on the type of epithelium and other elements in the microenvironment, the vagina, ectocervix, and endocervix are all susceptible to a variety of infections (viral, bacterial, and protozoan). Both *Candida *species and *Trichomonas vaginalis *can infect the stratified squamous epithelium of the vagina and ectocervix. *Chlamydia trachomatis *and *Neisseria gonorrhoeae *can infect the endocervical columnar epithelium. Both types of epithelium are susceptible to the herpes simplex virus. Numerous microorganisms can cause vaginal and cervical infections, and multiple illnesses might coexist in the same person, making it difficult to pinpoint the exact cause [5].

The symptoms brought on by pathological discharge include dyspareunia, a burning feeling, itching, and aberrant odour. The treatment of vaginal discharge frequently employs a syndromic strategy. The primary drawback of this strategy is an incorrect diagnosis and irrational use of numerous antimicrobials, which results in the emergence of drug-resistant strains that places a financial burden on the patient [6]. Simple laboratory procedures, such as Gram staining, wet mount, smell tests, and the use of direct microscopy can aid in identifying the etiological agent and defining the proper course of treatment thus preventing complications. 

Bacterial vaginosis, trichomoniasis and vulvovaginal candidiasis [7] are the three main etiological factors causing abnormal vaginal discharge. In addition to the reasons listed above, cytolytic vaginosis (also known as lactobacillus overgrowth syndrome or Doderlein's syndrome) should be taken into account as a possible cause of abnormal vaginal discharge. Cytolytic vaginosis is characterised by an excessive growth of lactobacilli, which results in the lysis of vaginal epithelial cells with symptoms like pruritus, dyspareunia, and vulvar dysuria [8]. Noninfective causes of vaginal discharge include atrophic vaginitis, foreign body, malignancy, contact dermatitis, or other mechanical or chemical irritation [9]. An intrauterine contraceptive device can also cause vaginal discharge related to chronic irritant cervicitis or endometritis.

The main sign of bacterial vaginosis (BV) is an offensive malodorous discharge. The proliferation of many facultative and anaerobic bacterial species is the main cause of it, which is prevalent in women who have multiple sex partners [10,11]. Severe itching and a white, curd-like discharge are two main features of vulvovaginal candidiasis. A significant amount of yellowish or greenish, occasionally foamy, discharge is a symptom of vaginal trichomoniasis [9].

Different forms of infections can be distinguished by the characteristics of aberrant vaginal discharge, such as its frequency, color, consistency, smell, and presence or absence of itching. Fever, pelvic soreness, and pelvic discomfort are warning signs of pelvic inflammatory disease [12]. Discovering patients who are at high risk for sexually transmitted infection, such as young women, those who have recently changed partners, those who engage in unprotected sexual activity, and those who have several partners, can be done by asking them about their sexual history. Given the widespread social stigma and common misconceptions about sexual health, it is crucial to elicit the patient's "agenda" and investigate their health views.

The aim of this study is to elucidate the cause of abnormal vaginal discharge among rural women and also to educate them about the practice of vaginal hygiene. If practised appropriately, it could prevent abnormal vaginal discharge and its systemic complications. Though our study used a simple microscope, it served a valuable purpose in the early management of vaginal discharge and the prevention of its complications in the community.

## Materials and methods

Study setting

The study was a cross-sectional study conducted in a rural health center of a medical college, in the rural part of Tamil Nadu, India, over a period of six months from February 2022 to July 2022.

Study population

After obtaining clearance from the Institutional Ethical Committee, Shri Sathya Sai Medical College and Research Institute (approval no. 725/2022), the study was initiated in February 2022. The study population comprised outpatients attending the primary care center with complaints of abnormal vaginal discharge. Pregnant and menopausal females were excluded.

Study methodology

The prevalence of vaginal discharge was 11.8% based on a study conducted by Durai V et al [13]. Based on that, the sample size calculated was 159. About 10% of the sample size was added to take care of any refusal to participate in the study and the total sample size arrived was 175. One hundred and seventy-five patients who satisfied the inclusion criteria were incorporated into the study at random after applying the criteria mentioned above. Prior consent was obtained. A detailed history was taken and a clinical examination was done. A thorough gynaecological examination was performed using a sterilised Cusco's speculum, which was inserted into the vagina to visualise the vagina and cervix. Any abnormalities or aberrant findings were documented. The abnormal vaginal discharge was then collected with three sterile swabs from the upper part of the posterior fornix. The amount, colour, character, and smell of the vaginal discharge were noted.

The pH was measured using litmus paper ranging from 2 to 10. Colour change was noted and correlated against the indicator. Out of the three swabs, the first was utilised for making a wet mount to assess the motility of *Trichomonas vaginalis*. The second was used to generate smears for gram staining and then to look out for clue cells. Finally, the third swab was used to perform a potassium hydroxide (KOH) mount for candida. 

Laboratory investigations

The following laboratory investigations were performed at the primary health centre [14]:

Candida

**Figure 1:**
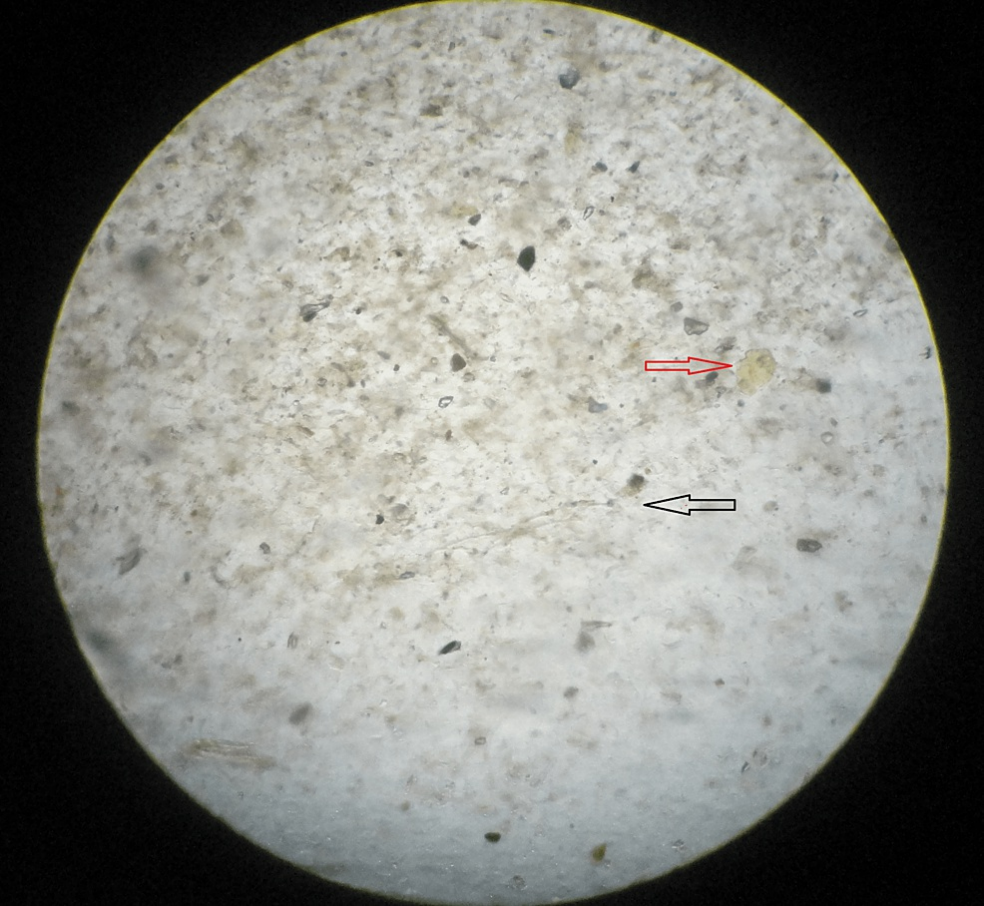
KOH smear shows vaginal epithelial cells (red arrow), pseudohyphae (black arrow) KOH: potassium hydroxide

**Figure 2:**
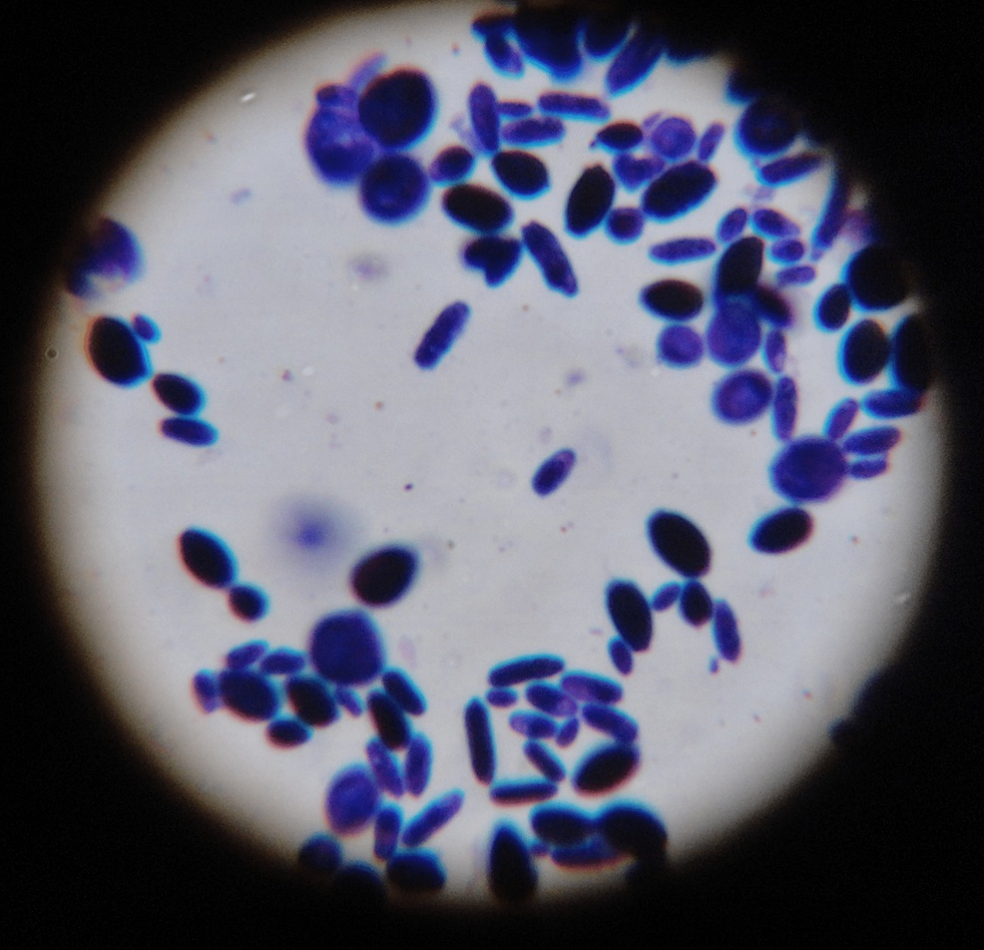
Gram stain showing yeast cells in vaginal smear

Potassium hydroxide (KOH) preparation: The vaginal secretions were taken on a clean glass slide and one drop of 10% KOH was added, covered with a coverslip, and mounted on a microscope. Candida was identified as round or oval budding yeast cells (Figure 1). In terms of Gram stain, Gram-positive budding yeast cells with pseudohyphae are shown in Figure 2.

Trichomonas vaginalis

Wet mount preparation: A drop of discharge was mixed with a drop of normal saline on a clean glass slide, covered with a coverslip, and viewed immediately under a microscope in 10X magnification. The characteristic twitching motility of *Trichomonads was* noted, as seen in Video 1.

**Video 1:** Video capture showing motile Trichomonads in vaginal smear

Bacterial Vaginosis

**Figure 3:**
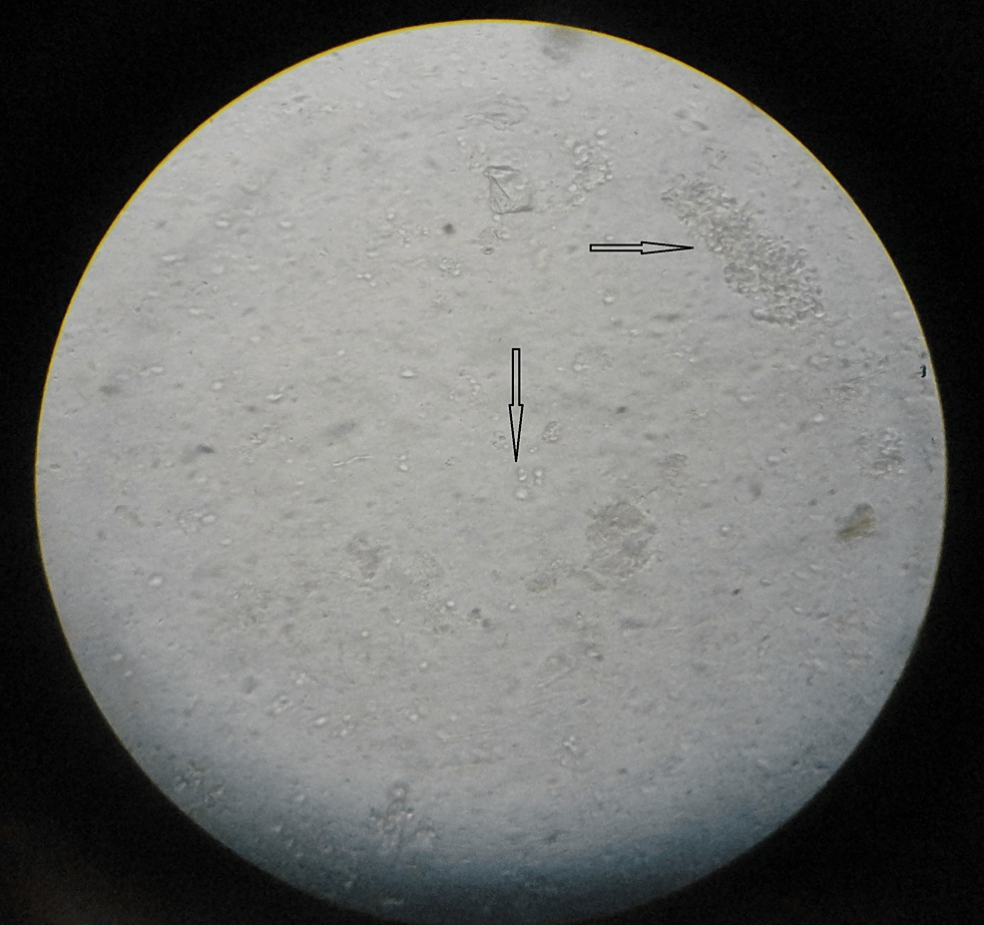
Saline mount showing clue cells (arrow pointing to the right) with inflammatory cells (arrow pointing downwards)

A whiff test/amine test was done as follows: a few drops of vaginal discharge were taken on a clean glass slide and two drops of freshly prepared 10% KOH solution were added to it. Both were mixed and smelled immediately. The wet film was examined under a microscope for the presence of clue cells - vaginal epithelial cells studded with numerous microorganisms, obscuring the border, thus exhibiting a fuzzy appearance (Figure 3).

**Figure 4:**
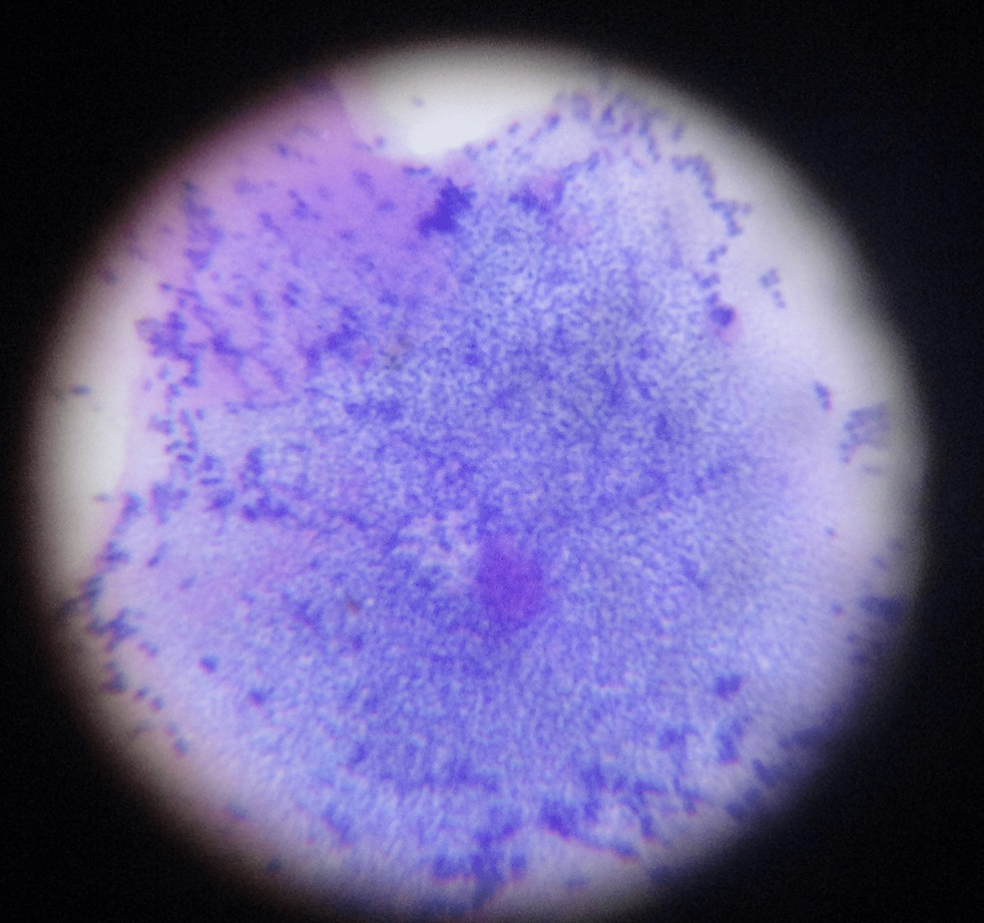
Gram stain of vaginal smear showing clue cells

Gram-stained smears were examined for the presence of altered vaginal flora in the form of Gram-negative coccobacilli studding vaginal epithelial cells instead of normally predominant Gram-positive lactobacilli (Figure 4).

Statistical analysis

The data collection sheet included the patient's demographic information, clinical features, examination findings, and laboratory investigation. Statistical analysis was carried out using SPSS software version 21 (IBM Corp., Armonk, USA). Descriptive statistics were described in terms of percentages. Inferential statistics was done using Chi-square and Fischer’s test. A p-value of less than 0.05 was considered to be statistically significant.

Ethical issues

The study was approved by the institutional ethical committee (IEC) before data collection. Informed written consent was obtained from the study participants before administering the questionnaire and performing clinical examinations.

## Results

The data was collected from a total of 175 women with a 100% response rate. The mean (SD) age of the study population was 34.8 (6.9) years. Almost half, 91 (52%), of the study participants were in the age group of 31-40 years. The data is represented in Table 1. 

**Table 1:** Age distribution of the study participants (n = 175) Mean 34.8 years, SD 6.9 years

Age group	Frequency (N=60)	Percentage
21-30	52	29.7
31-40	91	52
41-50	32	18.3
Total	175	100

Bacterial vaginosis was found in 74 (42.3%) patients and was the most common cause of abnormal vaginal discharge in our study participants followed by vulvovaginal candidiasis, 34 (19.4%). The other causes are listed in Table 2. 

**Table 2:** Etiology of abnormal vaginal discharge in the study participants (n = 175) BV: bacterial vaginosis; TV: *Trichomonas vaginalis; *VC: vulvovaginal candidiasis

Diagnosis	Frequency (N=60)	Percentage
Vulvovaginal Candidiasis	34	19.4
Bacterial Vaginosis	74	42.3
Trichomonas vaginalis	24	13.7
BV+TV	10	5.7
BV+VC	3	1.7
Non-specific	30	17.1
Total	175	100

It was found that about 27 (52%) cases in the 21-30 years age group were diagnosed as bacterial vaginosis. In the 31-40 years age group, 42 (46.1%) cases were diagnosed as bacterial vaginosis. The difference was found to be statistically significant with a significant p-value of 0.021. The data is represented in Table 3.

**Table 3:** Association between age and etiology of abnormal vaginal discharge BV: bacterial vaginosis; TV: Trichomonas vaginalis; VC: vulvovaginal candidiasis

Diagnosis	Age group			Total	Fisher’s Exact test
	21-30	31-40	>40		
Vulvovaginal Candidiasis	3 (5.7 %)	20 (22%)	11 (34.4%)	34 (19.4%)	0.021
Bacterial Vaginosis	27 (52%)	42 (46.1%)	5 (15.6%)	74 (42.3%)	
Trichomonas vaginalis	10 (19.2%)	10 (11%)	4 (12.5%)	24 (13.7%)	
BV+TV	6 (11.5%)	2 (2.2%)	2 (6.2%)	10 (5.7%)	
BV+VC	0 (0)	3 (3.2%)	0 (0)	3 (1.7%)	
Non-specific	6 (11.5%)	14 (15.4%)	10 (31.2%)	30 (17.1%)	
Total	52 (100%)	91 (100%)	32 (100%)	175 (100%)	

Among 175 women in our study, 59 (33.7%) were commercial sex workers (CSW) and 21 (12%) had a history of extramarital contact (EMC). About 95 (54.3%) of the study population had no significant (CSW/EMC) sexual behavior. Among the commercial sex workers who came with vaginal discharge, 31 (52.5%) had bacterial vaginosis and 18 (30.5%) had *Trichomonas vaginalis*. Among patients with extramarital contacts, 12 (57.1%) had bacterial vaginosis. The difference was found to be statistically significant with a highly significant p-value of 0.001. The data is represented in Table 4.

**Table 4:** Association between sexual behaviour and aetiology of abnormal vaginal discharge BV: bacterial vaginosis; TV: Trichomonas vaginalis; VC: vulvovaginal candidiasis

Diagnosis	Sexual behaviour			Total	Fisher’s Exact Test
	CSW	EMC	No risk behaviour		
Vulvovaginal Candidiasis	6 (10.2%)	3 (14.3%)	25 (26.3%)	34 (19.4%)	0.001
Bacterial Vaginosis	31 (52.5%)	12 (57.1%)	31 (32.6%)	74 (42.3%)	
Trichomonas Vaginalis	18 (30.5%)	2 (9.5%)	4 (4.2%)	24 (13.8%)	
BV+TV	4 (6.8%)	2 (9.5%)	4 (4.2%)	10 (5.7%)	
BV+VC	0 (0)	0 (0)	3 (3.1%)	3 (1.7%)	
Non-specific	0 (0)	2 (9.5%)	28 (29.5%)	30 (17.1%)	
Total	59 (100%)	21 (100%)	95 (100%)	175 (100%)	

Among 175 study participants, 29 (16.6%) had diabetes mellitus, six (3.4%) had HIV, and three (1.7%) had both diabetes and HIV. Among the patients with diabetes mellitus, 21 (72.4%) had vulvovaginal candidiasis. Among HIV patients, 4 (66.6%) had vulvovaginal candidiasis. The difference was found to be statistically significant with a p-value of 0.001. The data is represented in Table 5.

**Table 5:** Association between co-morbidities and abnormal vaginal discharge BV: bacterial vaginosis; TV: Trichomonas vaginalis; VC: vulvovaginal candidiasis; DM: diabetes mellitus

Diagnosis	Comorbidity				Total	Fisher’s Exact test
	DM	HIV	DM+HIV	No Comorbidity		
Vulvovaginal Candidiasis	21(72.4%)	4 (66.6)	3 (100)	6 (4.4)	34 (19.4)	0.001
Bacterial Vaginosis	2 (6.9%)	0 (0)	0 (0)	72 (52.5)	74 (42.2)	
Trichomonas Vaginalis	2 (6.9%)	2 (33.3)	0 (0)	20 (14.6)	24 (13.7)	
BV+TV	0 (0)	0 (0)	0 (0)	10 (7.3)	10 (5.7)	
BV+VC	0 (0)	0 (0)	0 (0)	3 (2.2)	3 (1.7)	
Non-specific	4 (13.8%)	0 (0)	0 (0)	26 (18.9)	30 (17.1)	
Total	29 (100)	6 (100)	3 (100)	137 (100)	175 (100)	

## Discussion

Throughout the study period, 175 individuals presenting with abnormal vaginal discharge were investigated. A prevalent health issue among women in the reproductive age group is vaginal discharge. Women frequently ignore it, whether it is severe or asymptomatic, which makes diagnosis more challenging.

Various parts of the world have different rates of pathogens in vaginal discharge [14-18]. Both pathological and physiological conditions can cause vaginal discharge. It is challenging to estimate the percentage of discharges that fall into each group. Despite the fact that many cases of abnormal vaginal discharge are not brought on by sexually transmitted infections (STIs), many treatable STIs can also manifest with this symptom.

The study population's average age (SD) in our study was 34.8 (6.9) years. Ninety-one (52%) study participants, or nearly half, were between the ages of 31 and 40. This is because the majority of women in this age range are sexually active. Similar outcomes were found in a study carried out in Salem, Tamil Nadu, by Venugopal S, et al [16]. Similar findings were also obtained by Al Quaiz JM in his Saudi Arabian study [17].

Bacterial vaginosis was discovered in 74 (42.3%) study participants as the most frequent cause of abnormal vaginal discharge, followed by vulvovaginal candidiasis in 34 (19.4%). In the 21-30 years age range, it was found that bacterial vaginosis was the diagnosis in roughly 27 (52%) cases; 42 (46.1%) patients in the 31-40 years age group had bacterial vaginosis as the diagnosis. Similar findings were made by Koumans et al [15], who discovered a 29.2% prevalence of BV. Similar to our study, Pawanarkar and Chopra's study [18] found that BV was widespread in 19% of women, making it the most frequent cause of vaginal tract infections.

Twenty-one (12%) of the 175 women in our study had a history of extramarital involvement (EMC), and 59 (33.7%) were commercial sex workers (CSWs). Thirty-one (52.5%) and 18 (30.5%) of the commercial sex workers had bacterial vaginosis and trichomoniasis, respectively. Twelve (57.1%) out of 21 of the patients with extramarital contact had bacterial vaginosis. Statistical analysis reveals the difference to be substantial, with a highly significant p-value of 0.001. In their study, Bradshaw CS et al discovered that bacterial vaginosis was linked to high-risk sexual activity indications such a new partner, more male partners in the past year, and more lifetime sexual partners (p<0 .05) [19].** **

In our investigation, diabetes mellitus was identified to be the most prevalent co-morbidity, observed in 29 (16.6%) patients, followed by HIV in six (3.4%). Twenty-one (72.4%) patients with diabetes mellitus who presented with vaginal discharge were found to have vulvovaginal candidiasis. Four (66.6%) HIV patients had vulvovaginal candidiasis as their diagnosis. These immuno-compromised conditions reduce the host's immune response and make them more susceptible to candida infections.

Our study is not without limitations. A cross-sectional study design allowed us to find only the association and not the causation between the effect and the cause. Owing to the diversified cultural characteristics, this study would have been exceptional if it had been performed in multiple centres to identify the various other causative agents of abnormal vaginal discharge and to initiate appropriate management.

## Conclusions

The most common cause of abnormal vaginal discharge was found to be bacterial vaginosis compared to vulvovaginal candidiasis and trichomoniasis. In a resource-limited setting, the usage of simple investigative tools aids in the diagnosis of a specific pathology. This helps in reducing the overuse of antibiotics, thus limiting resistance to them. There were significant associations between high-risk sexual behavior and the presence of co-morbidities with abnormal vaginal discharge. With a lack of response to antibiotics by these organisms, pertinent usage of antibiotics is warranted. Complications like cervical dysplasia, pelvic inflammatory disease (PID), and infertility pose a serious threat if these infections are left untreated.
